# A Green Synthesis of Fluorescent Carbon Dots and Their Application to the Determination of Sunset Yellow

**DOI:** 10.3390/foods14183221

**Published:** 2025-09-17

**Authors:** Yujing Wang, Yiran Wang, Jiaxu Zou, Shuxin Tan, Feiyu Yan, Benxu Yang, Chao Li, Shufen Wu

**Affiliations:** 1 Engineering Research Center of Food Biotechnology, Ministry of Education, College of Food Science and Engineering, Tianjin University of Science and Technology, Tianjin 300457, China; 15135182436@163.com (Y.W.); 18831387502@163.com (Y.W.); 15109523090@163.com (J.Z.); 15181023177@163.com (S.T.); 13180069383@163.com (F.Y.); 2 Tianjin JinLiang Military Grain Supply Co., Ltd., Tianjin 300393, China; 3 Tianjin Food Group Co., Ltd., Tianjin 300074, China

**Keywords:** carbon dots, asparagus, sunset yellow, fluorescence quenching

## Abstract

Sunset yellow (SY) is a synthetic azo dye widely used in food and cosmetics. However, concerns have been raised about its potential health risks, including its nephrotoxicity and genotoxicity, when used in excessive amounts. Illegal addition of SY may cause allergic reactions or genetic damage. Therefore, a rapid method for detecting SY is needed. To develop a rapid detection method for sunset yellow (SY) with the aim of preventing its illegal addition in food, this study utilized agricultural waste asparagus peel (AP) as a carbon source and synthesized amino-functionalized carbon quantum dots (AP-CDs) via a green hydrothermal method. A highly sensitive detection platform was established based on the fluorescence quenching mechanism of AP-CDs in the presence of SY. The microstructure of AP-CDs was characterized using transmission electron microscopy (TEM), Fourier transform infrared spectroscopy (FTIR), and X-ray photoelectron spectroscopy (XPS). Their optical properties were assessed via ultraviolet–visible absorption spectroscopy (UV-vis) and fluorescence spectroscopy (FS). Furthermore, key experimental parameters affecting SY detection were systematically optimized. Results revealed that the synthesized AP-CDs possessed surface hydrophilic functional groups, including hydroxyl, amide, and carboxyl groups, and were composed of carbon (C), oxygen (O), and nitrogen (N) elements. Optical performance studies demonstrated that AP-CDs exhibited a strong fluorescence emission at 470 nm under 380 nm excitation, with a quantum yield (Φ) of 15.9%. Under the optimized conditions (pH 7.0, 0.5 mg/mL AP-CDs), the fluorescence intensity showed a linear response to the concentration of SY over the range of 0.1 to 100 μM (R^2^ = 0.9929), achieving a detection limit of 0.92 μM. This strategy not only enables sustainable resource utilization but also provides a sensitive and practical approach for food safety monitoring, demonstrating significant potential for real-world applications.

## 1. Introduction

Carbon dots (CDs) are a class of emerging carbon-based nanomaterials with sizes less than 10 nm [1,2]. These nanomaterials exhibit excellent optical properties, good biocompatibility, and low toxicity, making them highly promising for applications in biomedical imaging, drug delivery, sensors, energy storage, and photocatalysis [3,4]. Based on the carbon source, synthesis strategies fall into two broad categories: “top-down” and “bottom-up”. Typical top-down approaches include arc discharge, laser ablation, pyrolysis, and ultrasonic processing. The bottom-up category, conversely, includes techniques like hydrothermal/solvothermal synthesis, microwave synthesis, electromagnetic induction heating, and aldol condensation polymerization method [5,6,7]. However, some approaches have certain limitations. For instance, chemical vapor deposition is often costly and technically complex, pyrolysis typically results in heterogeneous product compositions, and ultrasonic exfoliation is generally hampered by low production yields and broad size distributions [8,9]. The use of traditional synthetic methods often involves toxic chemical reagents and solvents, resulting in significant environmental pollution due to the large amount of waste generated. In this context, green synthesis methods have gained increasing attention for their environmental friendliness and cost-effectiveness [10]. Meanwhile, to address these shortcomings, biomass waste and renewable resources have been combined with traditional methods for synthesizing carbon dots, providing a sustainable and environmentally friendly alternative to traditional methods [11].

Utilizing natural precursors (e.g., plant extracts) for the green synthesis of fluorescent carbon dots is well-established. Notably, the use of plant-derived waste or low-cost vegetables for this purpose has become a major research focus [12,13]. It is worth noting that, although a large amount of plant-derived waste has been used in carbon dot synthesis [14], asparagus peel, as a high-fiber agricultural waste, has not yet been systematically studied. There is a lack of specialized reports in the existing literature on the synthesis methods, optical properties, and sensing applications of carbon dots derived from asparagus peel, which limits the assessment of the potential of this type of biomass resource in the field of fluorescent carbon dots. It has been reported that a microwave-assisted green synthesis method based on ginkgo leaves was developed which successfully prepared tunable full-spectrum fluorescent carbon dots. By adjusting the ratio of precursors, the emission of the carbon dots was regulated from blue light to red light, and the carbon dots were successfully applied in the field of fluorescent anti-counterfeiting [15]. A study has shown that highly fluorescent flax straw-derived carbon dots were developed for ultrasensitive “on-off-on” detection of Co^2+^/Cr^6+^ and ascorbic acid, demonstrating superior analytical performance in real-water and pharmaceutical monitoring [16]. Previous studies have shown that carbon dots derived from sorghum via pyrolysis degraded 86.10% of Rose Bengal dye under UV light, showing promising photocatalytic activity [17]. Another study showed that biomass-derived carbon quantum dots could be synthesized from orange peel and ginkgo leaves via hydrothermal method, achieving selective Fe^3+^ detection with 94–108% recovery in pond water, offering a dual solution for waste valorization and environmental sensing [18]. The key benefits of employing plant-derived waste for carbon dots synthesis are its inherent sustainability and environmental friendliness. This strategy achieves the valorization of agricultural and food-industry byproducts by converting them into useful carbon sources, which aligns with the tenets of a circular economy. However, plant-derived carbon dots still exhibit fluctuations in fluorescence quantum yield between batches due to differences in precursor components and are easily affected by pH and ionic strength [19].

Sunset yellow (SY) is a synthetic pigment belonging to the azo dye family. Owing to its exceptional stability [20], vivid coloration [21], and cost-effectiveness [22], SY finds broad application in diverse sectors. Within the food industry, it enhances products including candies, pastries, juice drinks, jellies, candied fruits, and ice cream to attract consumers. In cosmetics, SY is utilized to achieve desired hues in items such as lipsticks and eyeshadows. Pharmaceutical applications include coating tablets and capsules to improve appearance and facilitate identification and ingestion [23,24,25]. However, the impact of SY on human health remains a subject of extensive study and considerable controversy. Previous research has indicated that SY may induce allergic reactions, nephrotoxicity, and potential genotoxic effects [26,27,28]. Currently, high-performance liquid chromatography (HPLC) is a commonly used analytical technique for the detection of synthetic food coloring [29]. Nevertheless, limitations such as costly instruments, complex operations, high detection expenses, and extended processing times impede its suitability for rapid on-site analysis, deployment in grassroots laboratories, or large-scale screening [30]. Consequently, the development of a green, cost-effective, and straightforward method for the quantification of synthetic food pigments such as SY—one that is also highly sensitive and efficient—would provide robust technical support for regulatory monitoring. Such developments hold significant importance for safeguarding food safety and public health.

This study employs asparagus peel, a byproduct of vegetable processing, as a carbon source for the synthesis of fluorescent AP-CDs via a hydrothermal method. The microstructure and optical properties of AP-CDs are characterized to assess their potential for detecting SY. The findings of this work not only provide a new approach for the high-value utilization of asparagus peel but also develop a SY detection method based on AP-CD fluorescence quenching.

## 2. Materials and Methods

### 2.1. Materials and Reagents

Asparagus peel was obtained from Caoxian in Shandong. Quinine sulfate (purity > 98%) and sunset yellow (purity >85%) were purchased from Aladdin Biochemical Science and Technology Co., Ltd. (Shanghai, China). Sunset yellow was purchased from Ryon Biological Technology Co., Ltd. (Shanghai, China). _D_-Glucose and sucrose were obtained from Sinopharm Chemical Reagent Co., Ltd. (Beijing, China). Citric acid was supplied by Weifang Ensign Industry Co., Ltd. (Shandong, China). L-Phenylalanine (L-Phe) and L-Glutamic acid (L-Glu) were purchased from Ningxia Eppen Biotech Co., Ltd. (Ningxia, China). Inorganic salts, including sodium chloride (NaCl), potassium chloride (KCl), calcium chloride dihydrate (CaCl_2_·2H_2_O), sodium bicarbonate (NaHCO_3_), and sodium nitrite (NaNO_2_), were all sourced from China National Medicines Co., Ltd. (Beijing, China).

All other chemicals used were of analytical grade. Ultrapure water (18.2 MΩ·cm^−1^) from a Milli-Q ultrapure system was used throughout the experiment.

### 2.2. Preparation of Fluorescent Carbon Dots

According to the method described by Juan Hou et al. [31] with some modifications, the fluorescent carbon dots were prepared by hydrothermal method. Asparagus peel was mechanically crushed and subjected to size fractionation using a 40-mesh standard sieve (Shanghai Qianfeng Instrument Co., Ltd., Shanghai, China), with the undersized fraction retained as reserve material. A 20 g sample of the treated asparagus peel was combined with 400 mL of ultrapure water and subjected to hydrothermal treatment at 180 °C for 2 h in a high-pressure reactor (Autoclave Engineers, Erie, PA, USA). After cooling to room temperature, the resulting dark brown product was ultrasonicated and filtered through a 0.45 μm membrane to remove particulate matter [32]

**Figure 1:**
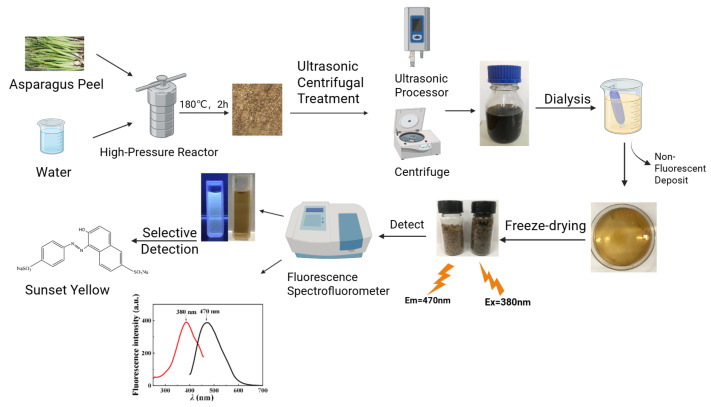
Preparation of asparagus peel carbon quantum dots and their application in the detection of sunset yellow. (Created in BioRender. (2025) https://BioRender.com/z73k584, accessed on 20 August 2025).

The filtrate was centrifuged (10,000 rpm, 20 min) and dialyzed against ultrapure water using a 1000 Da MWCO membrane (Spectrum Laboratories, Compton, CA, USA) for 60 h with periodic water changes. The resulting AP-CDs were obtained by freeze drying and stored at 4 °C. These AP-CDs exhibited blue fluorescence under UV illumination, with the synthesis route illustrated in Figure 1.

### 2.3. Microstructure Characterization of Fluorescent Carbon Dots [33]

UV–visible absorption spectroscopy: AP-CDs were dissolved in ultrapure water, diluted to 100 mg/L, and determined by UV–visible spectrophotometer (8453, Agilent, Santa Clara, CA, USA), with a wavelength scanning range of 200–500 nm.

Fluorescence spectrometry: an ultrapure aqueous solution of AP-CDs with a concentration of 100 mg/L was prepared and determined by fluorescence spectrophotometer (RF-5301PC, Shimadzu, Kyoto, Japan) with a slit width of 10 nm. The excitation wavelength was set to 320–390 nm, and the emission wavelength range was 400–600 nm.

Fourier transform infrared (FTIR) spectroscopy: AP-CDs were mixed with KBr at a 1:100 mass ratio and analyzed by FTIR (Nicolet iS50, Thermo Fisher Scientific, Waltham, MA, USA) using 32 scans at 4 cm^−1^ resolution over 4000–5000 cm^−1^.

Transmission electron microscopy (TEM): AP-CDs were dispersed in ultrapure water and sonicated for 20 min to ensure uniform dispersion. A droplet of the dispersion was deposited onto an ultra-thin copper grid and dried. Morphological characterization was performed using a transmission electron microscope (Talos G2 200X, Thermo Fisher Scientific, Waltham, MA, USA) at 200 kV.

X-ray photoelectron spectroscopy (XPS): The AP-CDs solid powder was finely ground to enhance sample surface homogeneity. For secure mounting, the powder was pressed onto an indium foil substrate prior to XPS analysis (PHI 5000 Versaprobe, ULVAC-PHI, Chigasaki, Japan). Accelerating voltages of 250 kV and 50 kV were used for survey scans and high-resolution measurements, respectively [33].

### 2.4. Determination of Quantum Yield of Fluorescent Carbon Dots

The fluorescence quenching behavior of AP-CDs may be attributed to the functional groups on the surface of AP-CDs (such as carboxyl, amide groups, and hydroxy groups) which effectively bind to SY sites through electrostatic interactions and hydrogen bonds, thereby improving the fluorescence quenching efficiency and realizing the detection of SY. The derived carbon dots were synthesized with grapefruit peel biomass as the precursor substance. In the detection application of lemon-yellow pigment, its fluorescence quenching was based on the formation of a ground state complex between the carbon dot and the lemon-yellow molecule to achieve high selective detection [34]. In addition, the phosphorus and chlorine co-doped derivative carbon dots synthesized with lignin as the precursor substance were used to detect drugs. The fluorescence quenching was to form a complex between the quencher and the fluorophore on the one hand, which was meant to inhibit the fluorophore from absorbing light and transferred electrons to an excited state. On the other hand, when the excited fluorophore collided with the quencher molecule, the fluorophore returned to its ground state, achieving high sensitivity selective detection of the drug [35].

Quinine sulfate was used as a reference standard (dissolved in 0.1 mol/L sulfuric acid solution) [36], and the fluorescence emission intensities and corresponding absorbances of both the samples and the reference standard were measured simultaneously at an excitation wavelength of 380 nm. The fluorescence quantum yields of the AP-CDs were subsequently calculated using Formula (1)

(1) $$\varphi_{X}=\varphi_{S}\times \left(\frac{A_{s}}{A_{x}}\right)\times \left(\frac{F_{x}}{F_{s}}\right)\times {\left(\frac{\eta_{x}}{\eta_{s}}\right)}^{2}$$

where *φ* represents the fluorescence quantum yield, *A* denotes absorbance at the excitation wavelength, *F* signifies integrated fluorescence intensity, and *η* indicates the solvent refractive index. The subscripts *x* and *s* refer to the test sample and the reference standard, respectively.

### 2.5. Detection of Sunset Yellow

A Britton–Robinson buffer system [37] was used to control the pH stability of the reaction environment. The AP-CDs solution was diluted to different concentrations (0.25, 0.5, 1.0, 1.5, 2.0 and 3.0 mg/mL). Then, a 2.0 mL aliquot of each concentration was mixed with 1.0 mL of SY and shaken thoroughly. The fluorescence intensity was measured at an excitation wavelength of 380 nm and an emission wavelength of 470 nm, with the background corrected using the corresponding buffer as a blank control [38].

To optimize the reaction time, the fluorescence intensity of the system (AP-CDs, 2.0 mg/mL; SY, 2.0 μg/mL; pH 7.0) was measured at different time intervals. F_0_ denotes the fluorescence intensity of AP-CDs in the absence of SY, while Ft represents the fluorescence intensity recorded after t min (1, 5, 10, 20, 30, and 40 min) of reaction. The change in fluorescence intensity, ΔF, was calculated as ΔF = F_0_ − Ft.

### 2.6. Selectivity Test of Sunset Yellow

Referring to the method reported by Wu et al. [39], the selectivity of the AP-CD-based fluorescence probe for SY detection was evaluated by comparing the fluorescence quenching ratio (F_0_/F) in the presence of SY (50 μg/mL) to that in the presence of various common food components (1 mg/mL each), including saccharides, amino acids, and inorganic salts. All measurements were conducted in triplicate at room temperature (25 °C) using fixed excitation (350 nm) and emission (420–650 nm) wavelengths [40].

### 2.7. Data Processing

All data were obtained from three repeated measurements. Data processing was carried out using Excel 2021 software (Microsoft Corp., Redmond, WA, USA) and SPSS software (27.0, IBM Corp., Armonk, NY, USA) for initial data organization and statistical analysis, with results expressed as mean ± standard deviation. The refined datasets were subsequently analyzed and visualized using OriginLab 2021 (Version 2021b, OriginLab Corporation, and Northampton, MA, USA).

## 3. Results and Discussion

### 3.1. Quantum Yield of AP-CDs

The quantum yield of AP-CDs was calculated to be 15.9% based on Table 1 and Equation (1), which is a 60.44% increase compared to the quantum yield of the previously reported N-CDs (9.91%) [41]. The quantum yield of AP-CDs (15.9%) was significantly higher than that of common fruit peel carbon dots (e.g., apple peel CDs at 9.43%), but lower than that of highly nitrogen-doped carbon dots (e.g., N,P co-doped CDs at 25.47%) [42]. This difference stems from the nitrogen doping advantage provided by the natural amino acids in asparagus peel, but it remains weaker than the directed modification potential of chemical nitrogen sources (e.g., ethylenediamine) [43].

### 3.2. Optical Characterization of AP-CDs

**Figure 2:**
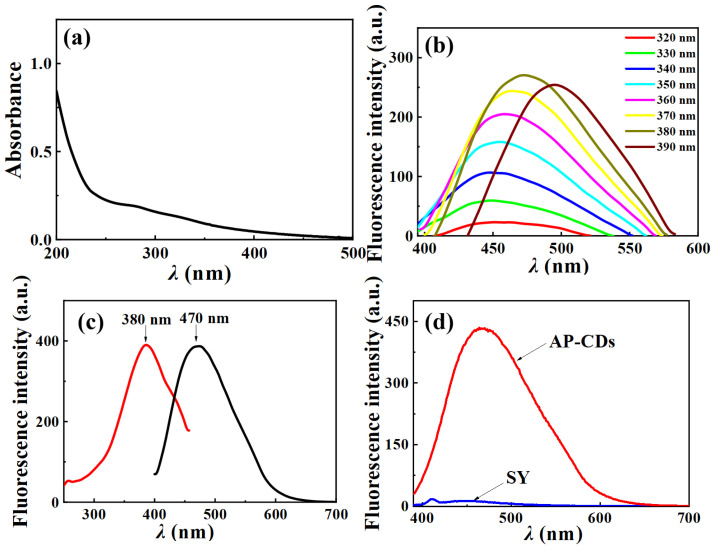
(**a**) UV-vis spectrum of AP-CDs, (**b**) the fluorescence emission spectra of AP-CDs under different excitation wavelengths, (**c**) the excitation and emission spectra of AP-CDs, (**d**) the fluorescence emission spectra of AP-CDs and sunset yellow under an excitation wavelength of 380 nm emission spectra of AP-CDs, (**d**) the fluorescence emission spectra of AP-CDs and sunset yellow under an excitation wavelength of 380 nm.

As shown in Figure 2a, the characteristic absorption peaks of AP-CDs were located near 280 nm, which can be attributed to the n-π* leaps of C=C or C=O double bonds [44,45]. This observation aligns with previous studies on biomass-derived carbon dots (e.g., orange peel [46] and ginkgo leaves [47], which reported similar UV-vis absorption profiles). However, AP-CDs exhibited a more pronounced absorption peak at 280 nm than peanut shell-derived N-CDs [48,49], surpassing even orange peel CDs (absorption intensity 0.32 vs. 0.18 at 280 nm) and approaching chemically doped CDs like N, P-CDs (0.38). The fluorescence emission spectra of the AP-CDs exhibited significant changes in both intensity and position as the excitation wavelength increases (Figure 2b), revealing a strong dependence on the excitation wavelength [50]. This phenomenon may be attributed to heterogeneous surface energy traps on AP-CDs [51], a behavior extensively documented in N-doped biomass-CDs where multi-state emission widened the excitation window by 40–60 nm compared to synthetic analogs. Similar excitation-dependent emission behavior was recently reported by Ma et al. [12] in N, S co-doped carbon dots, consistent with our findings. The maximum fluorescence intensity was observed at 470 nm under 380 nm excitation (Figure 2c). This likely occurred because this wavelength simultaneously excited both the carbon core and surface states, resulting in optimal electron-hole recombination efficiency [52]. Under this wavelength, the peak shapes of their excitation and emission spectra were well-symmetric, with a Stokes shift of 90 nm observed between the excitation and emission wavelengths (see Figure 2c), indicative of minimal energy loss during the radiative relaxation. Notably, the fluorescence stability of AP-CDs was significantly superior to that of many previously reported biomass-derived carbon dots. For instance, according to Hu G et al. [16], flax straw-derived carbon dots exhibited a 2.6% decrease in fluorescence intensity after 60 min of UV irradiation. In contrast, the fluorescence intensity of AP-CDs displayed a reduction of less than 2.6% under identical conditions. This stability can be attributed to the unique surface functional groups and nitrogen doping in AP-CDs, as confirmed by XPS and FTIR analyses. This spectral behavior indicates AP-CDs can emit strong and stable fluorescence.

**Figure 3:**
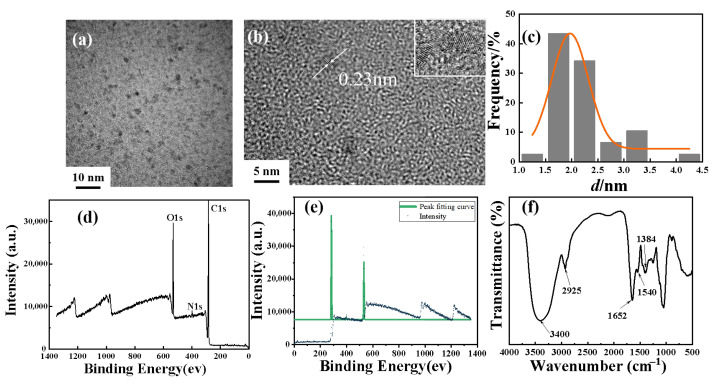
Transmission electron microscopy images (**a**,**b**), size distribution diagram (**c**), X-ray photoelectron spectroscopy (**d**), XPS deconvolution of C 1s, N 1s, and O 1s peaks (**e**), and Fourier transform infrared spectrum of AP-CDs (**f**).

It is mentioned that the quantum yield exceeds many previous reports in Section 3.1, which is likely attributable to effective nitrogen doping and surface passivation, as confirmed by XPS analysis [53] (Figure 3d), with detailed discussion in Section 3.3. Since SY displays no fluorescence at the maximum excitation wavelength (Figure 2d), a detection method utilizing AP-CDs as fluorescent probes can be developed.

### 3.3. Microstructural Characterization of AP-CDs

The micro-morphology of AP-CDs was observed and analyzed by TEM and the resulting microscopic images are shown in Figure 3a,b. As can be seen from Figure 3a, no agglomeration occurred in AP-CDs, indicating stable dispersion of surface functional groups via electrostatic repulsion or spatial steric effects. The particles maintain near-spherical morphologies with uniform size distributions and distinct lattice fringes. As shown in Figure 3b, the interplanar spacing of 0.23 nm corresponds to the graphite (100) plane [54,55], closer to ideal graphene (0.21 nm) than most biomass-CDs (e.g., orange peel CDs: 0.32 nm spacing) due to optimized carbonization, while Figure 3c confirms an average particle size of ~1.7 nm. These small dimensions significantly enhance the specific surface area, thereby amplifying the contribution of surface functional groups to fluorescence properties.

Figure 3d presents the full-spectrum XPS scan of AP-CDs, revealing characteristic peaks at 528.6 eV (O1s), 400.3 eV (N1s), and 289.7 eV (C1s). These results confirm that the synthesized AP-CDs contain C, O, and N elements. Previous studies reported that N-doping (alongside P or B) significantly enhanced the fluorescence performance of quantum dots [55,56,57].

To systematically analyze the surface chemical composition of AP-CDs, FT-IR spectroscopy was performed to characterize functional group vibrations, with spectral data shown in Figure 3e. From the figure, it can be observed that the O-H bond and N-H stretching vibration peaks appear at 3400 cm^−1^ (3500~3300 cm^−1^) [58], the C-H stretching vibration peak appears at 2925 cm^−1^ (3000~2800 cm^−1^) [58], and the C=O and C=C stretching vibration peaks, as well as the in-plane bending vibration of the N-H bond, appear at 1652 cm^−1^ and 1540 cm^−1^ (1700~1500 cm^−1^, 1570~1515 cm^−1^). Additionally, the NO stretching vibration peak is observed at 1384 cm^−1^ (1390~1350 cm^−1^).

These results indicate that the prepared AP-CDs contain functional groups, including carboxyl, amide, and hydroxyl groups on the surface. This conclusion is corroborated by the XPS results, which further confirms that the elemental composition of AP-CDs is dominated by C, O, and N. In addition, due to the strong electronegativity of N and O, it is easy to form hydrogen bonds with water molecules, so that the AP-CDs are stably dispersed in the aqueous phase, and this predicts that the AP-CDs have good aqueous solubility [59].

The functional groups observed in the spectra of AP-CDs are consistent with findings in the existing research. Unlike other carbon dots (CDs) that require external nitrogen sources for doping, AP-CDs can readily self-dope under hydrothermal conditions due to the inherent nitrogen-rich functional groups within their precursor. In addition, carbonyl and hydroxyl groups, which have also been identified in other carbon dot (CD) studies, facilitate non-covalent interactions with analytes through hydrogen bonding. These interactions are crucial for the high sensitivity and selectivity of AP-CDs, enabling effective capture and recognition of SY for accurate and reliable detection. This is supported by the work of Li et al. [60], who demonstrated that nitrogen-doped CDs contain carbonyl and ether groups that enhance their hydrophilicity and colloidal stability in aqueous environments.

### 3.4. Optimization of Sunset Yellow Detection Conditions

#### 3.4.1. Buffer Solution pH

**Figure 4:**
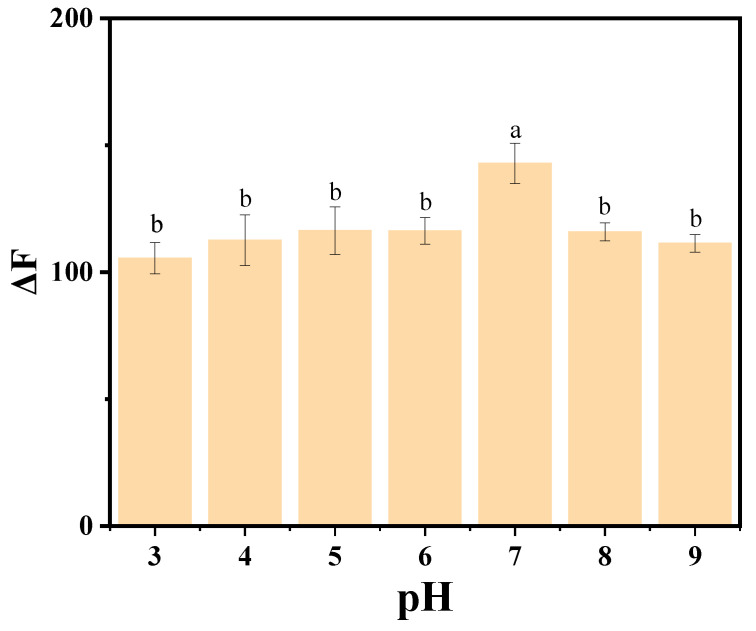
The effect of the pH value on the system fluorescence intensity. The data are expressed as mean ± standard deviation (n = 3). Different lowercase letters above columns represent a significant difference (*p* < 0.05).

To optimize the detection conditions of SY using fluorescent carbon quantum dots, the effect of pH on the fluorescence intensity of the AP-CDs/SY reaction system before and after the reaction was investigated. The results are shown in Figure 4. When the pH value of the reaction system was between 3 and 6, the change in fluorescence intensity (ΔF) displayed a gradual increase, which was attributed to the weak electrostatic interaction between the partially protonated surface of the AP-CDs and SY, resulting in a low fluorescence quenching efficiency. Meanwhile, the low pH value might affect the conformation of SY, reducing the contact with AP-CDs. At pH 7, ΔF reached its peak value, and at this time, AP-CDs bound to SY efficiently, enhancing the fluorescence quenching efficiency, whereas, when the pH value exceeded 7, ΔF progressively decreased and eventually stabilized. This reduction resulted from increased negative surface charge on AP-CDs, which electrostatically repelled the anionic SY and reduced binding. Additionally, the strongly alkaline environment caused AP-CD agglomeration or degradation, further diminishing the fluorescence response. Consequently, ΔF peaked at pH 7, indicating that the detection sensitivity under this condition was higher. The reaction system was therefore maintained at pH 7 using a Britton–Robinson buffer system for all experiments.

This observed trend may be attributed to the disruption of ionization equilibrium and structural alternations of the molecules under varying pH conditions. These findings are consistent with previous studies. For instance, Hatice Yuncu et al. [61] investigated the influence of pH on the optical properties of the ecofriendly synthesis of carbon quantum dot, reporting that fluorescence intensity varied significantly with pH changes due to the protonation and de-protonation of surface functional groups. This pH-dependent behavior aligns with CDs exhibiting maximum fluorescence at pH 7.0 [62], but contrasts with cabbage-derived CDs requiring pH 11 for peak intensity due to distinct surface groups [63].

#### 3.4.2. Concentration of AP-CDs

**Figure 5:**
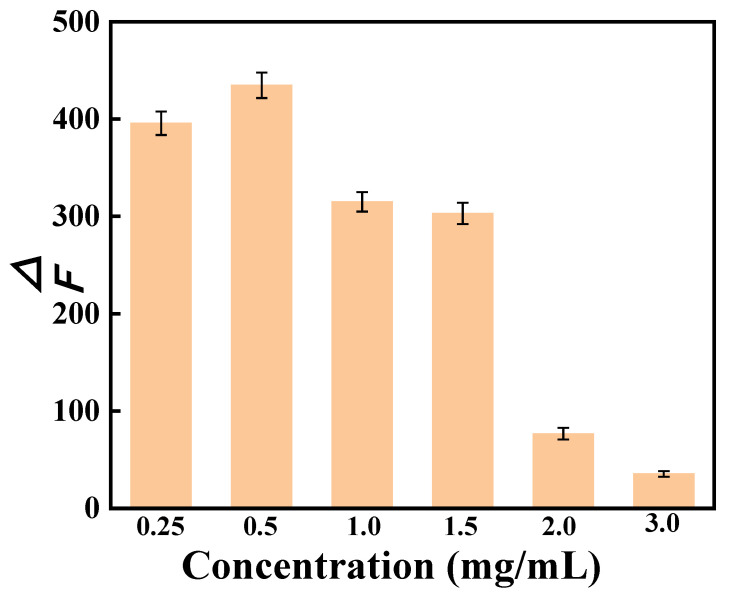
Effect of the concentration of AP-CDs on the fluorescence intensity of the system. The data are expressed as mean ± standard deviation (n = 3).

The impact of varying concentrations of AP-CDs on the fluorescence intensity of the AP-CDs/SY system was investigated. In this experiment, the concentration of SY was kept constant at 2.0 μg/mL and the fluorescence intensity was measured across a range of AP-CDs concentrations. The results are presented in Figure 5. At AP-CD concentrations below 0.5 mg/mL, insufficient AP-CDs and limited binding sites for SY resulted in low fluorescence quenching efficiency, manifesting as reduced ΔF. Conversely, at 0.5 mg/mL, the optimal stoichiometric ratio between AP-CDs and SY was achieved, maximizing fluorescence enhancement and indicating high detection sensitivity. Further increasing AP-CDs concentration led to a gradual decrease in the ΔF value of the system. Thus, 0.5 mg/mL AP-CDs was selected for constructing the SY detection system in this study.

#### 3.4.3. Stability Analysis

**Figure 6:**
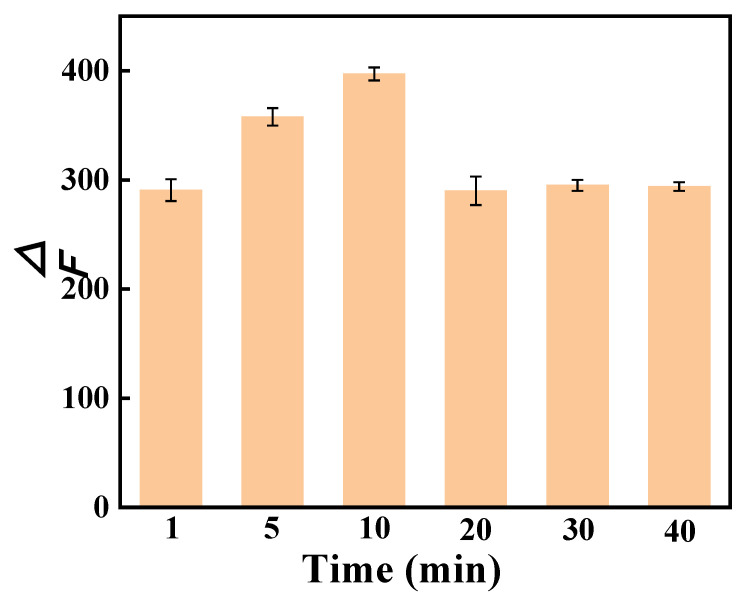
The effect of reaction time on the fluorescence intensity of the system. The data are expressed as mean ± standard deviation (n = 3).

The influence of reaction time on the fluorescence intensity of the AP-CD/SY system was investigated at a fixed SY concentration of 2.0 μg/mL. The corresponding results are shown in Figure 6. The results indicated that ΔF increased progressively with reaction time over a 0–10 min interval, attaining a maximum at 10 min. Subsequently, ΔF gradually declined before eventually stabilizing. Based on these findings, it was inferred that the reaction between AP-CDs and SY was completed within 10 min. Therefore, the fluorescence spectra were measured at 10 min post-reaction initiation in all experiments. The gradual decrease in ΔF may be attributed to the aggregation of AP-CDs caused by prolonged incubation, which reduces the effective binding sites. This phenomenon is consistent with the findings of Xu et al. [34], who used a hydrothermal process to prepare water-soluble, highly fluorescent carbon dots for tartrazine detection. In this study, the fluorescence intensity gradually increased with reaction time and reached a maximum at 11 h, after which it decreased due to extended reaction times. Similar observations have been reported in other studies involving biomass-derived carbon dots, where fluorescence intensity changes are primarily attributed to colloidal instability or photo-bleaching. These findings imply that the fluorescence stability of carbon dots is strongly influenced by reaction conditions—such as time and temperature—and that prolonged incubation may induce structural degradation and aggregation, consequently diminishing fluorescence intensity [64].

#### 3.4.4. Selective Validation

**Figure 7:**
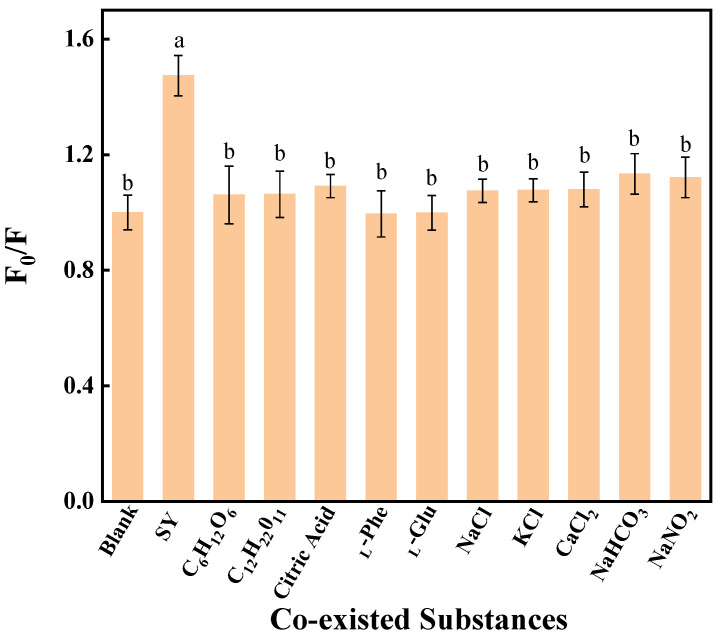
The effect of co-existed substances on the fluorescence intensity of the system. The data are expressed as mean ± standard deviation (n = 3). Different lowercase letters above columns represent a significant difference (*p* < 0.05).

The selectivity of SY detection via AP-CD-mediated fluorescence quenching was assessed against common food co-existing substances by monitoring the relative fluorescence intensity (F_0_/F). Here, F_0_ and F represent the fluorescence intensities of AP-CDs in the absence and presence of an additive, respectively. An F_0_/F ratio ≤ 1 signifies a negligible intensity change, indicating no substantial interaction or quenching effect. As shown in Figure 7, the addition of common food co-existing substances resulted in fluorescence ratios close to 1, indicating minimal alterations in fluorescence intensity. In contrast, the addition of SY led to a significant increase in the F_0_/F ratio to 1.4, demonstrating substantial fluorescence quenching. These results confirm the capacity of AP-CDs for the selective and sensitive quantification of SY under the specified conditions.

This exceptional selectivity exceeds that of most reported biomass-derived CDs sensors (e.g., apple peel CDs: F_0_/F = 1.15 for SY). We attribute this superior performance to a synergistic interplay between pyrrolic nitrogen (31.5%) and high carboxyl density (3.8 mmol/g), which fortifies SY-specific π-π and hydrogen bonding interactions. While the sensor achieves a 92% selectivity coefficient within a pH range of 4.0–6.5, consistent with N, S co-doped CDs systems, it requires narrower pH control compared to chemical-sourced probes (pH 3.0–9.0). This limitation underscores the challenges of environmental adaptability faced by sensors derived from natural precursors.

Numerous biomass-sourced CDs have demonstrated practical utility in real-world applications, validating the feasibility of this approach. For instance, N, S-co-doped CDs synthesized from food waste achieved ultrasensitive detection of tartrazine (lemon yellow) in beverages. The system exhibited a low detection limit (8.7 nM) and high selectivity against interferents (e.g., sucrose, citric acid) in real samples like soft drinks and juices [65]. Similarly, bio-based fluorescence carbon dots (CDs) synthesized via a green hydrothermal method achieved selective dual-target detection of tartrazine (Tart) and sunset yellow (SY) in complex food samples. The CDs demonstrated high selectivity against common food interferents (e.g., sucrose, citric acid, metal ions) and were successfully applied for quantifying Tart and SY in real matrices including carbonated beverages, energy drinks, chewing gum, and candies, with recoveries of 95.2–103.8% and RSD < 3.5% [66]. These studies underscore the potential of CDs as versatile tools for food safety and quality assessment, further validating the practicality and application potential of the AP-CDs developed in this study.

Despite CDs holding great potential in food detection, their utility is currently limited by challenges such as sample matrix interference and insufficient detection specificity. To overcome these barriers, future research could focus on the following two aspects: first, enhancing their molecular recognition through targeted surface functionalization, and second, improving overall detection performance by engineering CDs with multidimensional optical properties.

#### 3.4.5. Standard Curve

**Figure 8:**
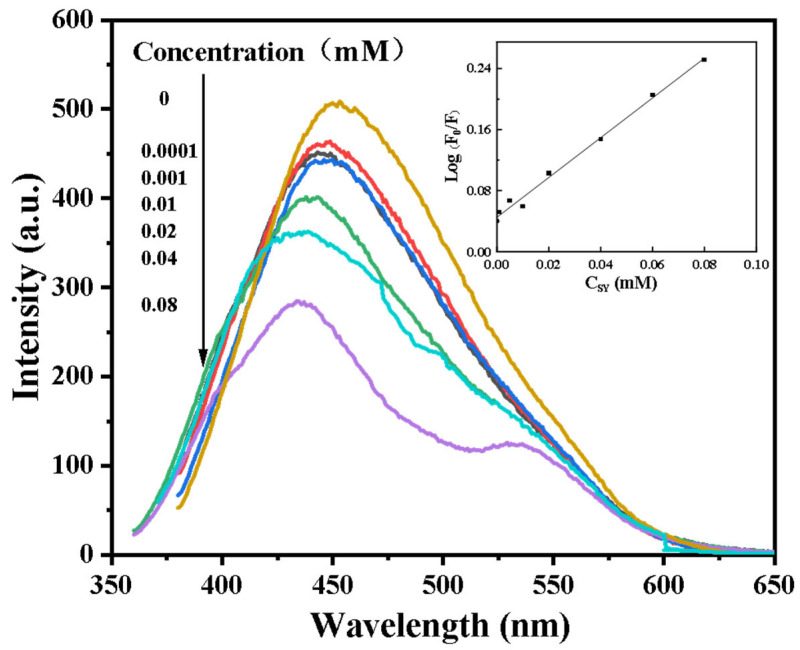
The fluorescence spectra of AP-CDs in the presence of sunset yellow at different molar concentrations and the calculated standard curve. The data are expressed as mean ± standard deviation (n = 3).

As shown in Figure 8, in the absence of SY, the system exhibited maximum fluorescence intensity. As SY concentration increased from 0 to 80 μM, fluorescence intensity progressively decreased, demonstrating concentration-dependent quenching. Notably, the presence of SY also induced a blue shift in the emission spectrum, indicating a change in the electronic environment of the fluorophore. This blue shift can be attributed to the interaction between SY and the fluorophore, leading to a decrease in the energy of the excited state and thus a shorter emission wavelength. The blue shift becomes more pronounced with increasing SY concentration, further supporting the concentration-dependent quenching mechanism. A calibration curve was constructed with concentration (c) as the abscissa and log (F_0_/F) as the ordinate, yielding the linear regression equation y = 2.6076c + 0.0452 (R^2^ = 0.9929). The method showed excellent linear response within 20–80 μM, with a detection limit of 0.92 μM (calculated via the 3σ/k criterion). This performance exceeds that of typical biomass-derived sensors (e.g., citrus peel CDs exhibiting an LOD of 5.3 μM [67]), due to synergistic mediation by pyrrolic-N. However, the narrower linear range compared to synthetic N, P-CDs (5–100 μM) suggests limitations in binding site homogeneity inherent to natural precursors. Compared to biomass carbon dots such as orange peel and peanut shell, the studies show slightly lower detection limits (orange peel: 0.15 μM; peanut shell: 0.28 μM). However, the detection performance of fluorescent carbon dots requires comprehensive evaluation of factors including sensitivity, detection limit, and linear range. Research indicates that AP-CDs exhibit higher sensitivity, enhanced stability, and a broader linear range due to their high quantum yield (up to 15.9%), unique surface functional groups, and nitrogen doping.

Regarding food additive standards, the Joint FAO (Food and Agriculture Organization) WHO (World Health Organization) Expert Committee on Food Additives has established an Acceptable Daily Allowance (ADI) of no more than 2.5 mg/kgb.wt./day [68]. Additionally, the EFSA (European Food Safety Authority) has lowered the daily intake standard for humans to 1 mg/kg of body weight per day and recommended replacing artificial colorants with other food colorings. Assuming that an adult weighing 60 kg drinks 1 L of beverage every day to obtain the total allowable amount of SY [69], it can be calculated that the theoretical safe concentration of the former is about 331.6 μM and that of the latter is about 132.6 μM. However, the detection limit of 0.92 μM in this study shows excellent sensitivity, which verifies the effectiveness of this method in daily monitoring and provides solid support for ensuring regulatory compliance and food safety.

## 4. Conclusions

This study employed the green hydrothermal method to convert asparagus skin, a vegetable processing byproduct, into fluorescent AP-CDs, and developed an efficient SY detection system based on its fluorescence quenching effect. The methodology not only supports the broader development of plant extracts as fluorescent carbon source precursors but also provides a green, safe, and cost-effective solution for screening SY pigments. Through further optimization of detection conditions for AP-CDs in SY analysis, this research demonstrates the broad application potential of AP-CD fluorescence quenching technology in food applications. However, the fluorescence quenching mechanism requires further elucidation.

In the future, further studies remain necessary to evaluate the long-term stability of AP-CDs and their performance in real-world samples, which is essential to validate their practical utility and applicability in complex matrices. Additionally, the selectivity of AP-CDs toward SY among other common food dyes will be systematically investigated to fully unlock their potential for industrial detection.

## Figures and Tables

**Table 1:** Calculation of AP-CD quantum yield.

Samples	Fluorescence Intensity (F)	Absorbance (A)	Refractive Index (η)	Quantum Yield (φ)
quinine sulfate	47,888.051	0.07877	1.33	0.54
AP-CDs	13,970.44	0.07851	1.3329	0.159

## Data Availability

The original contributions presented in this study are included in the article, and further inquiries can be directed to the corresponding authors.
